# Bacterial Reduction after Gutta-Percha Removal with Single vs. Multiple Instrument Systems

**DOI:** 10.22037/iej.v13i2.17505

**Published:** 2018

**Authors:** Felipe Xavier, Giselle Nevares, Luciana Gominho, Renata Rodrigues, Marcely Cassimiro, Kaline Romeiro, Diana Albuquerque

**Affiliations:** a *Department of Operative Dentistry and Endodontics, Dental College of Pernambuco, University of Pernambuco, Pernambuco, Brazil; *; b * Department of Endodontics, Federal University of Campina Grande, Campina Grande, Brazil;*; c * Molecular Microbiology Laboratory, Department of Endodontics, Faculty of Dentistry, Estácio de Sá University, Rio de Janeiro, Rio de Janeiro, Brazil*

**Keywords:** Endodontics, Enterococcus faecalis, Retreatment, Root Canal Instrumentation

## Abstract

**Introduction:**

The aim of this study was to evaluate the effectiveness of a reciprocating single-instrument system (Reciproc-REC) compared with combined continuously rotating multiple-instrument systems [D-Race (DR) and BioRace (BR)] in reducing *Enterococcus faecalis (E.f.)* after gutta-percha removal.

**Methods and Materials:**

Forty-six extracted human maxillary incisors were prepared and contaminated with *E.f. *strain (ATCC 29212) for 30 days. The samples were obturated and randomly divided into two experimental groups for gutta-percha removal (*n*=23): a REC group (R50) and a DR/BR group (DR1, DR2 and BR6). A standardized irrigation with 0.9% saline solution was performed. Root canal samples were taken with paper points before (S1) and after (S2) the removal of gutta-percha to establish bacterial quantification by culture. The time required for gutta-percha removal was also recorded. Positive and negative control groups (*n*=6) were used to test bacterial viability and control asepsis, respectively. Data were analysed using *t*-Student and one-way ANOVA tests (5% margin of error).

**Results:**

The mean percentage of bacterial reduction was significantly higher in DR/BR group (84.2%) than in REC group (72.3%) (*P*<0.05). The mean time for obturation removal was 74.00 sec in REC group and 107.53 sec in DR/BR group (*P*<0.05).

**Conclusion:**

The combined continuously rotating multiple-instrument system was more effective in reducing bacteria after the removal of gutta-percha than the single-instrument system. None of the tested systems was able to completely eliminate root canal infection after gutta-percha removal. Thus, additional techniques should be considered.

## Introduction

Endodontic treatment is highly successful, with a clinical success rate of over 92% [1]. However, even properly treated canals with well-established protocols can result in failure, and the main etiological factor is intraradicular presence of microorganisms [2]. Seeking for elimination or control of the infection, endodontic retreatment is the first treatment option. It initially involves removal of the filling material and reaching proper working length (WL) followed by cleaning and shaping of the root canal [3, 4].

Some nickel-titanium (NiTi) mechanical endodontic instruments were specially developed for removing the root filling material [5]. D-RaCe (DR) retreatment system (FKG Dentaire, La Chaux-de-Fonds, Switzerland) has proved to be efficient for this purpose [6] and it is composed of DR1 and DR2 instruments. The DR1 instrument (30/0.10) is specifically designed for gutta-percha removal in the cervical third and has an active working tip to promote initial entry into the filling material. The DR2 instrument (25/0.04) has a non-active tip to minimize operative errors and is used to reach the WL. After using the DR system, it is recommended that the final root canal shape should be achieved using additional instruments, such as the ones in the BioRace (BR) system (FKG Dentaire, La Chaux-de-Fonds, Switzerland). Both systems should be used with a continuous rotating motion and are designed with a triangular cross section without radial lands, and with sharping cutting edges.

Recently, NiTi instruments developed for shaping the root canal, have been tested in retreatment [7] and the Reciproc (REC) system (VDW, Munich, Germany) has achieved effectiveness in the removal of gutta-percha [8]. The REC system is available in different tip and taper sizes including R25 (25/0.08), R40 (40/0.06) and R50 (50/0.05), and was designed for the root canal thorough instrumentation with a single instrument in a reciprocating motion, *i.e*., movements alternating in clockwise and counter clockwise directions [9]. The instrument has an S-shaped cross section along its active part, with sharp cutting edges and a positive cutting angle, and there are no radial lands [10]. In retreatment cases, it was previously described that after the removal of the most coronal part of the root canal filling by Gates-Glidden or ultrasonic tips, the single-instrument REC should be used to remove all filling material and to complete the root canal preparation [11]. Several studies had evaluated the efficacy of REC system in removing root canal filling material [12-14]. However, no *in vitro* study has evaluated the efficacy of the REC system on bacterial reduction after the removal of gutta-percha. 

The purpose of this study was to compare the bacterial reduction after gutta-percha removal from the root canals contaminated with *Enterococcus faecalis (E.f.)* by using REC and DR/BR instrument systems. The effective time needed for the removal of gutta-percha was also recorded. The null hypothesis tested was the lack of significant differences in the effectiveness of REC and DR/BR systems for the parameters evaluated.

## Materials and Methods

The sample size was based on a previous study that observed the antimicrobial effectiveness on *E.f.* during endodontic retreatment [15]. A minimum size of 21 samples per group was required using the test of equal means (*t*-Student; Minitab Statistical Software 16.1, Minitab Inc., URL: www.minitab.com) with *α*=5%, power of 80% or upper and ratio of 1.00.

***Specimen selection and preparation***

This study was reviewed and approved by the Research Ethics Committee of the University of Pernambuco (31649114.7.0000.5207). Extracted human single-root maxillary incisors with lengths ≥ 20 mm and fully formed apices were selected. After radiographic examination, teeth with previously treated canals, pulp calcification or internal resorption were excluded. The crowns were reduced in height to achieve an overall length of 20 mm and endodontic access was performed. A glide path was established using a size 20 hand K-File instrument (Dentsply Maillefer, Ballaigues, Switzerland). Only teeth with this instrument adjusted with resistance to the apical foramen were selected. The total sample consisted of 58 roots. All root canals were prepared with BR0 (25/0.08) in the cervical third and BR1 (15/0.05), BR2 (25/0.04), BR3 (25/0.06), BR4 (35/0.04), and BR5 (40/0.04) up to the WL established at 1 mm short of the apical foramen. Irrigation was performed with a total volume of 12 mL of 2.5% sodium hypochlorite (NaOCl). After cleaning and shaping, smear layer was removed with 2 mL of 17% EDTA. Finally, the root canal was irrigated with 2 mL of 2.5% NaOCl. As previously described, NaOCl was inactivated with 10% sodium thiosulfate [16]. The root canals were filled with brain heart infusion (BHI) broth (Difco Laboratories, Detroit, MI, USA) and immersed in the same solution. Agitation for 1 min with an ultrasonic bath was performed to release entrapped air and allow penetration of BHI into root canal irregularities. The teeth were sterilised in an autoclave for 20 min at 121^°^C and kept at 37^°^C in the incubator for 24 h to check the efficacy of the sterilisation procedures. No microbial growth was observed in any of the tested specimens.

***Bacterial contamination and initial sample procedures (S1)***

The teeth were numbered and randomly placed (http://www.random.org) in two experimental groups (*n*=23), and positive (*n*=6) and negative (*n*=6) control groups. All procedures were conducted in a laminar flow chamber. In the negative control group, no contamination was induced and the teeth were submerged in sterile BHI until they were filled. A suspension of *E.f.* (American Type Culture Collection 29212) was prepared and standardised to tube 1 on McFarland scale and injected into the root canal in experimental and positive control groups. The teeth were incubated at 37^º ^C for 30 days, and the root canal contents were replaced every two days with fresh BHI broth. After the contamination period, the crown and external root surface were disinfected with 3% hydrogen peroxide, 2.5% NaOCl, and 10% sodium thiosulfate. The root canal was rinsed with 1 mL of sterile 0.9% saline solution (NaCl) to remove unattached cells. An initial bacteriological sample (S1) was obtained using five absorbent sterile paper points (size 40) inserted in WL for 1 min each. The points were stored in tubes containing 1 mL of saline. The samples were 10-fold serially diluted in saline (up to 10^-2^). Afterwards, aliquots of 10 μL were plated onto Mitis-Salivarius agar plates (Difco Laboratories, Detroit, MI, USA) and incubated at 37^º ^C for 48 h in order to calculate bacterial counts in colony-forming units (CFUs) based on known dilution factors. 

***Root canal filling***

Root filling was performed using a lateral condensation technique. A master gutta-percha cone (40/0.04) was fitted to WL and laterally condensed with a finger spreader and accessory gutta-percha cones (averaging 3-5 accessory cones per canal). The gutta-percha cones were cut to the level of the cementoenamel junction (CEJ) and vertically compacted. The pulp chamber was filled with fresh BHI and the roots submerged in BHI broth and incubated at 37^º ^C for 3 days.

***Bacterial samples after removal of gutta-percha (S2)***

After disinfection, the apical foramen was sealed with cyanoacrylate and the pulp chamber was irrigated with 3 mL of 0.9% NaCl. In both groups, the removal of gutta-percha was considered complete when it was no longer visualised between the cutting blades and when the canals exhibited smooth walls. All instruments were discarded after a single use in both groups. The total time needed to remove the gutta-percha and reach the WL was counted in sec. The time taken to irrigate, change, and clean the instruments was excluded. 

**Reciproc Group (REC)**: A size 1 Gates-Glidden drill (Dentsply Maillefer, Ballaigues, Switzerland) was used in the cervical third (4 mm) to create space inside the bulk of the gutta-percha to REC instrument. The Gates-Glidden was removed and cleaned, and the canal was irrigated with 4 mL of 0.9% NaCl for 60 sec. The R50 instrument (50/0.05) was used in reciprocating motion in the middle and the apical thirds. The R50 was introduced into the root canal until resistance was felt, and three forward and backward movements were performed with slight apical pressure. After 3 pecking motions, the instrument was removed from the canal and cleaned. The root canal was irrigated with 4 mL 0.9% NaCl for 60 sec. Then, root canal was explored up to WL using a size 20 K-file. These steps were repeated until the R50 reached WL. Finally, the canal was irrigated with 4 mL of 0.9% NaCl for 60 sec. A total volume of 12 mL of 0.9% NaCl was used. Bacteriological samples (S2) were obtained using paper points (size 50) as described in S1, and were also processed for culture analysis.

**D-Race and BioRace Group (DR/BR)**: The main bulk of filling material was removed by using the DR1 instrument (30/0.10; 1000 rpm and 1.5 N.cm) in the cervical third (4 mm) and the DR2 (25/0.04; 600 rpm and 0.7 N.cm) in the middle and apical thirds. After each instrument, the root canal was irrigated with 4 mL 0.9% NaCl for 60 sec. The root canal was explored up to WL using a size 20 K-file. The canal was then prepared; using the BR6 instrument (50/0.04; 500 rpm and 1 N.cm) in the WL. Afterwards, the root canal was explored up to WL using a size 20 K-file and irrigated with 4 mL 0.9% NaCl for 60 sec. All the instruments were used in a continuous rotating motion. After three forward and backward movements, performed with slight apical pressure, the instruments were removed from the canal and cleaned. Irrigation was performed in the same manner and with the same volume as for the REC group. The criteria to confirm removal of gutta-percha and the method for bacteriological sample collection samples (S2) also followed the same pattern as for the REC group. 

In the negative control group, the removal of gutta-percha was performed in the same way as REC (*n*=3) and DR/BR (*n*=3) groups to control asepsis. No attempt to remove gutta-percha was performed on the positive control group in order to test bacterial viability after filling procedures. In this group, the teeth were grooved in the buccolingual direction using a diamond disc and cleaved longitudinally with a rongeur. The gutta-percha was removed from the root canal and transferred to tubes containing 1 mL of sterile saline solution. In addition, bacteriological samples were taken from the dentinal surface using paper points and transferred to tubes containing saline solution, and were immediately processed for the evaluation of CFU counts as described above.

***Statistical analysis***

Log transformation of each CFU/mL count was performed, and statistical tests were applied. The *F*-test (ANOVA) with Bonferroni adjustment was used for intragroup analysis. Intergroup analysis and comparison of the total time needed to remove gutta-percha were performed using Student’s *t*-test. Verification of the hypothesis of equality of variances was performed using Levene's *F*-test and the normality hypothesis by means of the Shapiro-Wilk test. The margin of error used in the statistical tests was 5.0%.

## Results

No significant difference, between the REC and the DR/BR groups in the mean bacterial amount before filling procedures (S1), was found (*P*>0.05). 

Both techniques were able to significantly reduce the number of bacteria in the root canal after the removal of gutta-percha (S2) (*P*<0.05). The mean bacterial reduction after gutta-percha removal was greater in DR/BR group (84.2%) compared to REC group (72.3%) (*P*<0.05). The mean time required for gutta-percha removal was significantly higher in DR/BR group than REC group (*P*<0.05). Table 1 shows the log CFU/mL of *E.f*. before and after the removal of gutta-percha as well as the time recorded. 

The aseptic condition during the experiment was confirmed by the absence of bacterial growth in the uncontaminated samples. The positive control group confirmed bacterial viability after root canal filling.

**Table 1:** Mean (SD) of bacterial quantification (log CFU mL^-1^) before (S1) and after removal of gutta-percha (S2) and time taken to remove gutta-percha

	**S1**	**S2**	**Time (s)**
**Group (N)**	**Mean (SD)**	**Mean (SD)**	**Mean (SD)**
**REC (23)**	4.88 (0.66)	4.13 (0.75)	74.00 (22.72)
**DR/BR (23)**	4.59 (0.41	3.43 (0.62)	107.53 (41.37)

## Discussion

In analysing the bacterial reduction after gutta-percha removal from the root canals contaminated with *E.f., *the REC single-instrument system showed significantly lower results compared with the combined use DR and BR systems in the present study (*P*<0.05). Therefore, the null hypothesis was rejected for the studied parameter.

The effectiveness of bacterial reduction in root canals is directly related to mechanical instrumentation [16]. In REC group, filling material removal was performed using a Gates-Glidden drill in the cervical third [8]. In the middle and apical thirds, only R50 instrument was used to simultaneously remove filling material, clean, and shape the root canal [11]. As gutta-percha forms a mechanical barrier, its presence could have had more interference in the REC instrument performance. A previous study investigated the incidence of deformation on REC instruments after its clinical use and concluded that it was more frequent in retreatments than in primary ministrations [11]. Structural changes to the instrument during preparation can interfere with its cutting capacity, making it less efficient at cleaning. In DR/BR group, the DR system was specifically designed for retreatment and DR1 instrument had an active working tip for gutta-percha removal in the cervical third. Sharing the workload between multiple instruments may have favoured improved efficacy as well as greater bacterial reduction in DR/BR group. 

The apical third is the most critical portion requiring cleaning in retreatment procedures [17]. The presence of bacteria in this section is directly related to persistent infection [18]. The initial apical diameter of the upper incisor in WL can vary from 0.30 to 0.45 mm [4]. In the present study, the removal of gutta-percha was performed up to 0.50 mm diameter, similar to previous work [19]. Although the final shaping of the root canal was concluded with the same tip size in both groups, it was not possible to standardize the tapers. R50 instrument (50/0.05) is more tapered than BR6 instrument (50/0.04). It was expected that the greater is the cutting of dentin and the apical diameter enlargement, the higher is the reduction in the amount of bacteria [20]. Nevertheless, the results indicated the opposite. A previous clinical study compared the disinfection efficacy in retreatment between REC and a multiple instrument system [21]. An important difference between the tested systems in the previous study can be identified: the apical diameter of the REC instrument (25/0.08) is considerably larger than the apical diameter of the multiple-instrument system (20/0.07) tested. However, the results showed that a multiple-instrument system had similar results compared with REC system. Similarly, in the present study, the taper showed no influence on the results. 

Regarding kinematics, the REC single-instrument system featured a specific motor that performed the reciprocating motion. In DR/BR group, all instruments were used in continuous rotating movement. It was argued that the continuous rotation motion produced a constant flow of debris in the coronal direction and the reciprocating motion had a trend for debris to be displaced apically rather than moved coronally [12, 22]. From this perspective, the REC instrument would favour a large bacteria load inside root canal, meeting the found results. However, the influence of kinematic remains controversial and further studies are necessary to confirm these findings [23]. 

In this study, both systems tested were effective at reducing the amount of bacteria after the removal of gutta-percha (*P*<0.05). However, none of the samples showed 100% bacterial reduction, which was in agreement with previous studies that showed how difficult it was to completely eliminate bacteria after chemo-mechanical preparation [16, 24]. NaOCl is the most widely used irrigant solution in endodontic treatments owing to its effective antimicrobial activity and the ability to dissolve organic tissues [25]. This solution was not used in the present study due to the aim of comparing only the mechanical impact of the tested systems without the influence of antibacterial activities. For the same reason, solvent and sealer were not used [15, 26]. Evaluation of contaminated root canals through bacterial culture is clearly defined and* E.f. *was used as a bacteriological marker [15, 16, 27]. *E.f. *can play an important role in persistent endodontic infections because of its high resistance to endodontic procedures and its adaptation to aggressive environments [18, 28]. 

Although it is not a determining factor for system choice in retreatment procedures, clinicians search for a faster and safer way to successfully prepare root canals. Thus, the time taken for gutta-percha removal was recorded. In the present study, it was shown that the single-instrument system was faster than the tested multiple-instrument systems. Similar findings have been reported in previous study [8]. Thus, the null hypothesis was rejected for this parameter (*P*<0.05).

## Conclusion

Even though neither system could completely eliminate microorganisms from the root canal after the removal of gutta-percha, the multiple-instrument DR combined with BR system had better performance for bacterial reduction compared with the single-instrument REC system. Additional techniques should be considered to enhance the cleaning of root canals when single-instrumentation system is intended.
